# UPLC-MS/MS Based Identification and Quantification of a Novel Dual Orexin Receptor Antagonist in Plasma Samples by Validated SWGTOX Guidelines

**DOI:** 10.3390/toxics11020109

**Published:** 2023-01-23

**Authors:** Muzaffar Iqbal, Abdullah Alshememry, Faisal Imam, Mohd Abul Kalam, Ali Akhtar, Essam A. Ali

**Affiliations:** 1 Department of Pharmaceutical Chemistry, College of Pharmacy, King Saud University, Riyadh 11451, Saudi Arabia; 2 Department of Pharmaceutics, College of Pharmacy, King Saud University, Riyadh 11451, Saudi Arabia; 3 Department of Pharmacology and Toxicology, College of Pharmacy, King Saud University, Riyadh 11451, Saudi Arabia; 4 College of Pharmacy, King Saud University, Riyadh 11451, Saudi Arabia

**Keywords:** Lemborexant, insomnia, abuse, UPLC-MS/MS, SWGTOX, DORA

## Abstract

Lemborexant (LEM) is a novel dual orexin receptor antagonist (DORA), recently approved for the treatment of insomnia. As with other DORAs, LEM has potential of abuse and therefore placed in Schedule IV class by the United States Drug Enforcement Administration (USDEA). In this study, a sensitive and accurate UPLC-MS/MS assay was developed for the quantification of LEM in human plasma sample using losartan as an internal standard (IS). The chromatographic separation was performed by using gradient elution of mobile phase, comprising of 10 mM ammonium acetate and acetonitrile with a flow rate of 0.3 mL/min. An Acquity UPLC BEH C_18_ (1.7 μm, 2.1 × 50 mm) column was used for separation of LEM and IS by maintaining the oven temperature of 40 °C. The electrospray ionization in positive mode was used for sample ionization. The precursor to product ion transition of 411.12 > 175.09 (qualifier) and 411.1 > 287.14 (quantifier) was used for detection and quantification of LEM, respectively, in multiple reaction monitoring mode. Being a drug of abuse, the assay was validated according to “Scientific Working Group for Toxicology” (SWGTOX) guidelines, including limit of detection (LOD), limit of quantification (LOQ), precision and bias, calibration model, interferences, carry-over effects, matrix effects, and stability parameters. The LOD and LOQ of the assay were 0.35 and 1.0 ng/mL, respectively. The linear range was between 1–300 ng/mL with correlation coefficient of ≥0.995. The method was also cross validated in rat plasma samples with acceptable ranges of precision and accuracy before its application for pharmacokinetic study in rats.

## 1. Introduction

Insomnia is a most common sleep–wake disorder, affecting 30–50% of the adult population across the globe [1]. Benzodiazepines and sedative/hypnotics are the most commonly used pharmacological intervention for the management of insomnia. However, their use has been now restricted due to their adverse sleep related behaviors and cognitive/psychomotor impairment [2,3]. Orexin-1 and orexin-2 receptors (OX1R and OX2R), which are apparently expressed in various regions of brain, are recently considered as novel target for the treatment of insomnia [4]. Lemborexant (LEM) is the second approved dual orexin receptor antagonist (DORA) for the treatment of adult patients with insomnia as per the United States Prescribing Information (USPI) [5,6]. It has fast association and dissociation from the OX1R and OX2R in compared to other DORA, and therefore sleep can be achieved quickly and maintained throughout the night while avoiding next morning sleepiness or residual effects [7]. Moreover, it has been reported that LEM has a low propensity to impair next-day functioning among healthy subjects and the subjects suffering with insomnia [8]. The recommended dose of LEM for insomnia disorder is 5 mg to 10 mg daily before going to sleep. In various randomized clinical controlled trails, LEM significantly improved the sleep onset and sleep maintenance by approved dose and without producing residual morning sleepiness [9,10]. Recently, acute cognitive effects of LEM have been also reported in recreational sedative patients [11].

LEM is rapidly absorbed after oral administration of tablet form in humans with peak plasma concentration (C_max_), which was achieved within 1–3 h. The mean C_max_ and area under-curve (AUC_0–24 h_) increased slightly less than in proportion to dose after administration of 2.5 to 75 mg of LEM and the extent of accumulation was 1.5 to three-fold across the dose range at steady-state level. The volume of distribution for LEM is high (1970 L) with clearance rate of 32.8 L/h. The half-life (T½) is 15–17 h with 94% plasma protein binding [6]. It is mainly excreted through the feces (≈57.4%) and urine (≈29.1%), with around 1% as in unchanged form. It is lipophilic in nature, primarily metabolized by CYP3A4 enzyme. The C_max_ and AUC of LEM were increased by 1.4- to 1.6 time and 3.7- to 4-time by co-administration with itraconazole or fluconazole (strong to moderate CYP3A inhibitor). Similarly, its C_max_ and AUC were reduced by 90% when co-administered with rifampin (strong CYP3A inducer). Therefore, it is recommended to avoid the concomitant administration of LEM with strong or moderate inhibitors or inducers of CYP3A enzyme [12].

LEM is appeared to have similar abuse potential profile to suvorexant and zolpidem and therefore placed in Schedule IV controlled substance [13]. Being a drug of abuse, and also predominantly metabolized by CYP3A4 enzyme, a sensitive bioanalytical assay of LEM is necessary for the testing of drug of abuse in forensic toxicology, therapeutic drug monitoring, and to check or avoid any pharmacokinetic interaction. Until now, only one LC-MS/MS assay has been identified in the literature for the determination of LEM in human plasma, which was applied to an ex vivo protein binding study [14]. The purpose of our study was to develop a UPLC-MS/MS method for determination of LEM in human plasma. Due to abuse potential of LEM, the assay was validated by following the “Scientific Working Group for Toxicology” (SWGTOX) guidelines so that it could also be used for forensic laboratory testing in futuristic study [15]. Assay validation of schedule IV (suvorexant, eluxadoline, lorcaserin) controlled substances has been previously reported by our laboratory by following SWGTOX guidelines [16,17,18,19]. For proof of applicability, the validated method was successfully applied by analyzing rat plasma samples to support a pharmacokinetic study.

## 2. Materials and Methods

### 2.1. Chemicals and Reagents

**Figure 1:**
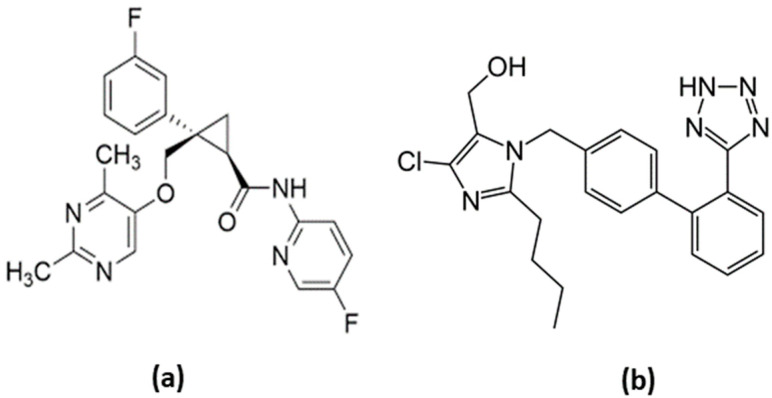
Chemical structure of LEM (**a**) and losartan (**b**).

LEM (purity; ≥99.0%) was purchased from “Beijing Mesochem Technology Co. Ltd. Beijing, China”. Losartan, used as internal standard (IS, purity > 98%) was from “Amriya Pharmaceutical Industries, Cairo, Egypt” (Figure 1). The HPLC grade methanol, acetonitrile (ACN) and ethyl acetate were purchased from “Fisher Scientific Limited, Leicestershire, UK”. The AR grade of ammonium acetate and dimethyl sulphoxide (DMSO), were procured from “Loba Chemie Pvt. Ltd. Mumbai, India”. The ultrapure deionized water dispensed by “Milli-QR Gradient A10R, Millipore, Moscheim Cedex, France” was used for aqueous solution preparation.

### 2.2. Instrumentation and Chromatographic Conditions

The UPLC-MS/MS system composed of an Acquity triple quadrupole (TQD) mass spectrometer with Acquity H-Class UPLC system (Waters^®^ Corporation, Milford, MA, USA). An electrospray ionization (ESI) probe operated in positive mode was used as ion source for sample ionization. The detection and quantification were performed under multiple reaction monitoring (MRM) mode using precursor to product ion transition of 411.12 > 175.09 as qualifier ion and 411.12 > 287.14 as quantifier ions for LEM. Quantifier-to-qualifier ion ratio was expected to be within 20% of those in QC samples. The optimized capillary voltage was 0.53 kV, while source and desolvation temperature were 150 °C and 350 °C, respectively. Ultrapure nitrogen (flow rate: 650 L/h) was used as desolvation gas and argon (0.17 mL/min) for collision gas, respectively. The optimized compound specific parameters are presented in Table 1.

The chromatographic separation of LEM and IS were achieved on Acquity UPLC BEH^TM^ C_18_ column (2.1 × 50 mm; 1.7 µm). The mobile phase comprising of 10 mM ammonium acetate (solvent A) and ACN (solvent B) was pumped in gradient mode at 0.3 mL/min of flow rate. The gradient condition of mobile phase used for sample separation is presented in Table 2. The column temperature was fixed to 40 ± 5 °C, whereas the auto-sampler temperature was 15 ± 5 °C during analysis. The variations of retention times for both LEM and IS were acceptable within ± 2%. The volume of each injection was 5 µL, and the total run time for each analysis was 4 min. The MassLynx software (Version 4.1) with Target Lynx^TM^ program was used to acquired and process all experimental data, respectively.

### 2.3. Stock Solution, Calibration Standards (CSs) and Quality Controls (QCs) Sample Preparation

The stock solution of LEM and IS were prepared by dissolving their requisite amount in DMSO and methanol, respectively, to achieve 1 mg/mL concentration. The stock solution of LEM was further diluted with ACN: water (50:50, *v*/*v*) to prepare working solutions for CSs. The blank plasma matrix was fortified with these working solutions to achieve eight CSs of 1, 2.84, 9.45, 31.5, 63, 126, 210, and 300 ng/mL. Similarly, QCs samples were prepared by fortifying the plasma matrix with working solutions to achieve concentrations of 3, 50, and 250 ng/mL and were treated as low (LQC), middle (MQC), and high (HQC) QC concentration, respectively. The stock solution of IS was diluted with ACN: water (50:50, *v*/*v*) to prepare a solution of 4 µg/mL concentration. All aqueous solutions were stored at 2–8 °C, while the fortified plasma matrix samples were placed in −80 °C during valid period.

### 2.4. Sample Extraction Procedure

In 150 µL of fortified plasma sample, 15 µL of IS (4 µg/mL) was added except the blank sample and vortexed each for 30 s. Thereafter, 1 mL of the ethyl acetate was added into each sample and again vortexed followed by cold centrifugation at 10,500× *g* at 4 °C. Then 800 µL of the supernatant organic layer was transferred to a fresh 1.5 mL capacity Eppendorf tubes. All the tubes were placed into sample concentrator and dried for 45 min. The remaining residue in tubes were reconstituted with 150 µL of pure acetonitrile (ACN) and 5 µL of this was injected into the UPLC-MS/MS for analysis.

### 2.5. Assay Validation

The validation was carried out as per the international parameters set by the SWGTOX in the Standard Practices for Method Validation in Forensic Toxicology [15]. The parameters included for evaluation were: limit of detection (LOD), limit of quantification (LOQ), interferences, calibration model, precision and accuracy, carry-over effects, recovery and matrix effects, dilution integrity, and stability studies.

#### 2.5.1. LOD and LOQ Determination

The LOD was considered as the lowest concentration of the calibrator for which signal-to-noise ratio (S/N) of the qualifier MRM transition was 3. It was determined by analyzing decreasing concentration of LEM to establish the lowest possible concentration that can be distinguished reliably from the limit of blank and the concentration at which detection was feasible. The LOQ was considered as the lowest concentration of calibrator with S/N ratio of 10 for the qualifier MRM transition. Furthermore, the LOQ should be the lowest concentration that can be quantified with acceptable precision and accuracy with a relative standard deviation (RSD) of <20%.

#### 2.5.2. Interference Studies

Interferences of endogenous substances form matrix was evaluated by analyzing the blank matrices which obtained from 10 different sources. The responses in blank matrices were compared with LOD and LOQ responses of the assay.

#### 2.5.3. Calibration Model

An appropriate calibration model is necessary for accurate and reliable quantitative determination. The linear regression using least square method was used to establish the calibration model of this assay. For this, eight different concentrations of CSs samples were analyzed by five replications in different run. The linearity was determined by plotting the calibration curve between the area ratios of analyte and IS versus nominal concentration of CSs. The coefficient of correlation (*r*) for the calibration curves should be ≥0.99. Further weighing factor of 1/X, 1/X^2^ and none were used to adjust the best fitting of the curve.

#### 2.5.4. Carry-Over Effects

The carry-over effects were evaluated by analyzing the blank plasma matrices, injected in triplicate just after the highest concentration of CSs sample. No significant peaks (≥20% of LOQ) should be observed in blank matrices samples to ensure the assay free from carry-over effects.

#### 2.5.5. Precision and Bias

Precision and bias studies have been evaluated concurrently by using LOQ and all three QC samples. It was measured in pooled fortified matrix using five replicates for each concentration in three batches for over three consecutive days. Precision is expressed as the relative standard deviation (%RSD) and was determined by calculating the mean and standard deviation of the response for each concentration. The bias is expressed as relative error (%RE), which was calculated by measuring the percentage difference in the calculated values for each concentration in compared to the nominal concentration divided by nominal concentration. Both intra- and inter-day variation in the precision and bias were determined and their acceptable criteria were ≤20% and ±20%, respectively.

#### 2.5.6. Matrix Effects

The enhancement or suppression of LEM ionization due to presence of co-eluting substances in matrices were evaluated by post extraction addition approach method. This approach is also known as quantitative method as amount of ionization or enhancement is assessed. For this, two different set of samples were prepared. Set one consists of neat solution of all three QC concentration levels, while set two consists of post extracted six different lot of matrix fortified with all three QC concentrations. The average area of each post extracted samples was calculated and compared with neat solution area to evaluate the ion suppression/enhancement effects. Same procedure was followed for IS matrix effects determination. The average suppression/enhancement effects must be ≤25%, and the %RSD value should not be >15% to ensure the assay is free from matrix effects.

#### 2.5.7. Assay Recovery

The recovery of LEM and IS were evaluated in plasma matrix at all three QC concentration in six replicates. For this, two sets of samples were prepared. One set consisting of plasma matrix fortified with QC concentration before extraction, while the other set of samples was fortified after post extraction. The percentage difference was calculated to determine the % recovery of LEM and same procedure was followed for IS recovery determination.

#### 2.5.8. Stability

This was designed to address the stability of LEM in plasma matrix at different storage conditions and sample processing procedure during laboratory operation. It was performed by analyzing plasma matrix fortified by two QC (LQC, HQC) concentrations. The short-term stability was evaluated by putting the samples for 8 h at ambient temperature before processing, while the autosampler stability was evaluated by analyzing the processed samples after keeping them in autosampler plate for 24 h at 15 °C temperature. The freeze-thaw stability was evaluated by analyzing the fortified QC samples after completion of three times freezing and thawing cycle. The long-term stability of the samples was performed by analyzing the fortified QC samples stored at −80 °C for 60 days.

### 2.6. Pharmacokinetic Study in Rats

The developed and validated assay was applied to a single dose pharmacokinetic study in rats. Male Wistar albino rats (weighing 250–300 gm) were received from “Animal Care Centre, College of Pharmacy, King Saud University, Riyadh KSA”. The animal experiment was performed in accordance with the guidelines of the “Experimental Animal Care and Use Committee of College of Pharmacy, King Saud University Riyadh”, and the experimental protocol was approved by “The Research Ethics Committee, King Saud University”, (Approval No. KSU-SE-22-17, dated 24/03/2022). After 12 h of fasting, rats were given LEM (10 mg/kg, i.g. dissolved in CMC), and blood samples (≈400 µL) were collected from the retro-orbital plexus into the heparinized tubes at 0, 0.25, 0.5, 1, 2, 4, 12 and 24 h). Plasma samples were harvested by centrifuging the blood at 4500× *g* for 8 min at 4 °C and kept at −80 ± 2 °C till the analysis was conducted. The pharmacokinetic parameters C_max_, T_max_, AUC, T½, mean residence time (MRT), and elimination rate constant (K_el_) were calculated by non-compartmental model using WinNonlin Software (version 4.0.1).

## 3. Results and Discussion

### 3.1. Mass Spectrometric Condition Optimization

**Figure 2:**
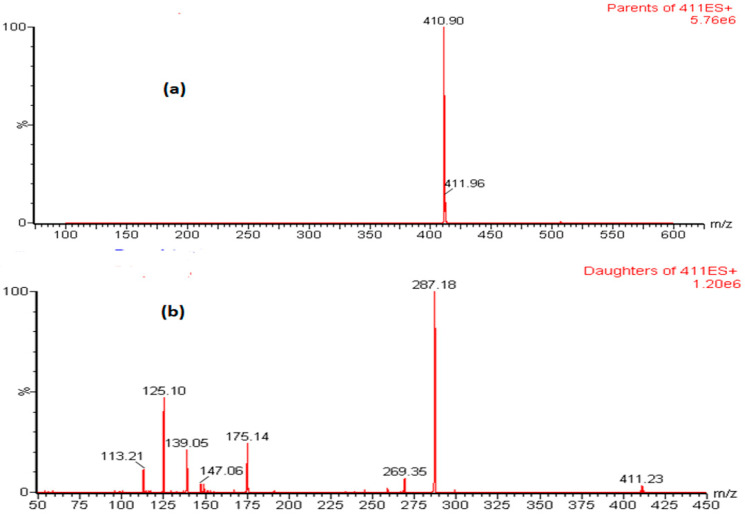
Representative LEM mass spectra of precursor ion (**a**) and precursor to product ion (**b**) in ESI positive mode.

Optimization of mass spectrometric condition was initially performed by standard solution tuning using IntelliStart method. MRM mode was used to carry out the quantitative analyses to achieve high selectivity and sensitivity. An aqueous standard solution of LEM and IS (500 ng/mL) were infused in both positive and negative mode by using combined flow system. It was observed that the ion intensity of LEM and IS were more intense in positive mode as compared to the negative mode. During fragmentation processing, the more abundant product ion of LEM was selected for quantitation (quantifier ion) and the less abundant product ion as qualifier for confirmation. Further mass spectrometric (general and molecule specific) parameters were optimized to achieve maximum possible ion intensity as presented in Table 1. The precursor and precursor to product ion transition spectra of LEM in ESI positive mode are well represented in Figure 2.

### 3.2. Chromatographic Condition Optimization

Due to hydrophobic nature of analyte, Acquity CSH and BEH column of different size (2.1 × 50 and 2.1 × 100 mm) with common particle size (1.7 µm) were tried for separation of analyte and IS. The result with Acquity BEH column of 2.1 µm × 50 mm size was better among them and was selected for chromatographic elution optimization. Initially, mobile phase comprising of ammonium acetate, formic acid with organic modifiers of methanol and ACN were tried for chromatographic elution in isocratic mode. Although ammonium acetate together with ACN produced better separation, both analyte and IS were eluted within 0.65 min of time, which reflects non-proper retention of molecules in stationary phase. Therefore, we switched to gradient elution mode, and it produced better separation of both with best resolution as described in Table 2. Usually, analyte labeled stable isotope is best choice for IS in order to minimize the difference in extraction recovery and matrix effects. Unfortunately, deuterated form of LEM is not commercially available in market and therefore we have tried here some common and easily available molecules with same ionization pattern and elution properties. In this regard, losartan produced better separation with optimized chromatographic condition and column and therefore selected as IS of this method.

### 3.3. Optimization of Sample Extraction Procedure

Optimization of sample extraction procedure is an important step for development of a reliable and reproducible bioanalytical assay. An ideal procedure should be simple with easy step, inexpensive, high recovery, and low matrix effects. Initially, protein precipitation method by using ACN, methanol, and its combination were tried. Although the recovery was satisfactory with ACN, the peak intensity was not stable, and the sensitivity was low, which requires further drying and reconstitution step. Then, liquid–liquid extraction was tested by using different organic solvents of dichloromethane, ethyl acetate, n-hexane and diethyl ether. Among these extracting solvents, the recovery with ethyl acetate was higher than others and, hence, it was selected as extracting solvent. Although the mean recovery of this assay (≈74%) was lower than previous reported method [12] by solid phase extraction (SPE), it is an expensive procedure and is of limited availability regarding that setup in maximum laboratory.

### 3.4. Method Validation

#### 3.4.1. LOD and LOQ

In this assay, LEM was detected and quantified down to 0.35 and 1 ng/mL, respectively, with the acceptable S/N ratio and was considered as LOD and LOQ of this assay. Moreover, the determined precision and bias for LOQ were within the acceptable limits (≤20%) as mentioned in SWGTOX guideline [13].

#### 3.4.2. Interference and Selectivity Studies

**Figure 3:**
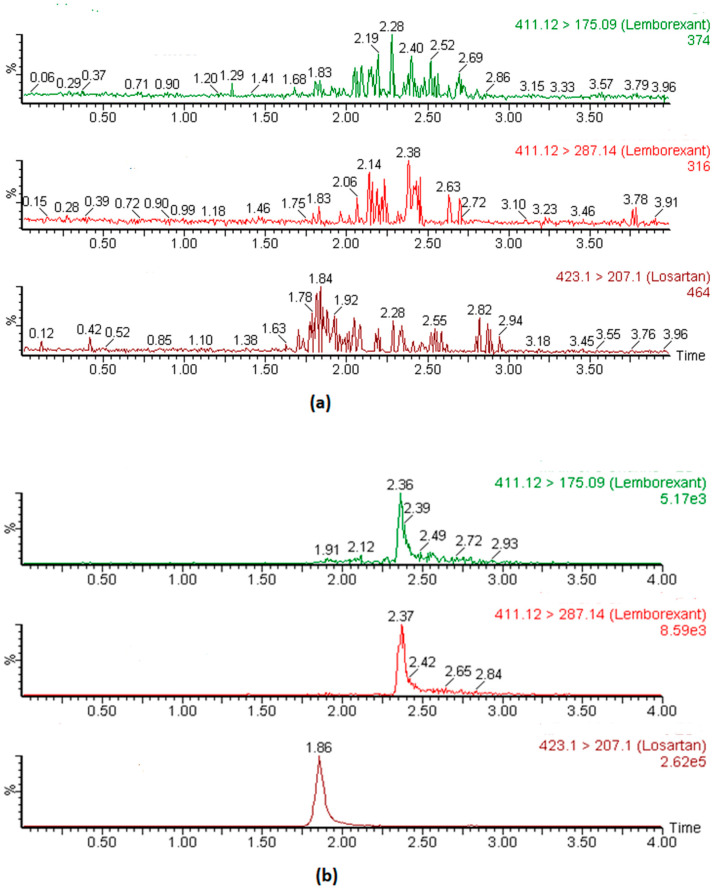
Representative MRM chromatogram of LEM and IS in blank plasma (**a**) and plasma fortified at LOQ level concentration (**b**). [411.12 > 175.09 → Qualifier ion; 411.12 > 287.14 → Quantifier ion; 423.1 > 207.1 → IS].

No significant interfering peaks were observed in the blank plasma chromatograms of all tested transition channels of analyte and IS. The extracted ion chromatograms of blank plasma and plasma spiked at LOD and LOQ concentration are depicted in Figure 3. These results confirm that the method is selective and specific for the analysis of LEM in plasma matrices.

#### 3.4.3. Calibration Model

The calibration curves plotted between area ratio (analyte/IS) versus nominal concentration of LEM were linear over the CSs range of 1.0–300 ng/mL using the propose least square method. The mean value of coefficient of correlation for calibration curves (*n* = 5) was 0.995 ± 0.002. The weighing factor (1/X^2^) has shown the best linear fit with lowest bias and was used for back calculation of concentration of the CSs. The deviation in the back calculated concentration of all CSs were found within the acceptable limit of ±15% of the nominal concentration.

#### 3.4.4. Carry-Over Effects

No significant peaks were found in the processed blank plasma matrices, which were analyzed just after the highest CS concentration (300 ng/mL). These results confirmed that the proposed method is free from carry-over effects and expected concentration of analyzed samples were accurate and reliable.

#### 3.4.5. Precision and Bias

The precision and bias results for LOQ and all three QC_s_ (LQC, MQC and HQC) concentrations processed in human plasma matrix are displayed in Table 3. The mean value of intra- and inter-day precision (% RSD) were ≤11.49 and ≤9.35%, respectively. The measured mean value of intra- and inter-day bias were ranged in −10.03% to 6.99% and −9.12% to 10.74%, respectively. All of these data ranges were within 15% of the limit, indicating acceptable bias and precision according to the SWGTOX guideline.

#### 3.4.6. Recovery and Matrix Effects

The extraction recovery and matrix effects results for LEM and IS are displayed in Table 4. As evident form the results, the overall mean recovery of LEM form plasma matrix was 73.9% with 5.68% of RSD, using ethyl acetate as extraction solvents. Although the % recovery is lower than the previous reported SPE method, the results were consistent and concentration independent between all three QC concentration [12].

During matrix effects evaluation, ion suppression effects were observed with MQC (93.3%) and HQC (87.4%) concentration, while ion enhancement effects (106.8%) were observed with LQC concentration, indicating minimal ion suppression/enhancement effects. The overall mean value was 95.8% and were under the limits of SWGTOX guideline [13].

#### 3.4.7. Stability

The stability of LEM fortified in plasma matrix using LQC and MQC concentration at different anticipated conditions are presented in Table 5. The results demonstrated that LEM is stable, up to 8 h after keeping the sample at bench top position before processing, in processed plasma samples after three freeze/thaw cycles, in processed plasma samples stored in autosampler up to 24 h, and after processing the fortified plasma samples stored for two months in deep freezer (−80 °C). It is concluded that the plasma samples can be stored in deep freezer (−80 °C) up to two months from their collection time to analysis. The aqueous standard solutions of LEM and IS were also stable for 15 days at refrigerator temperature.

### 3.5. Application in Pharmacokinetic Study in Rats

**Figure 4:**
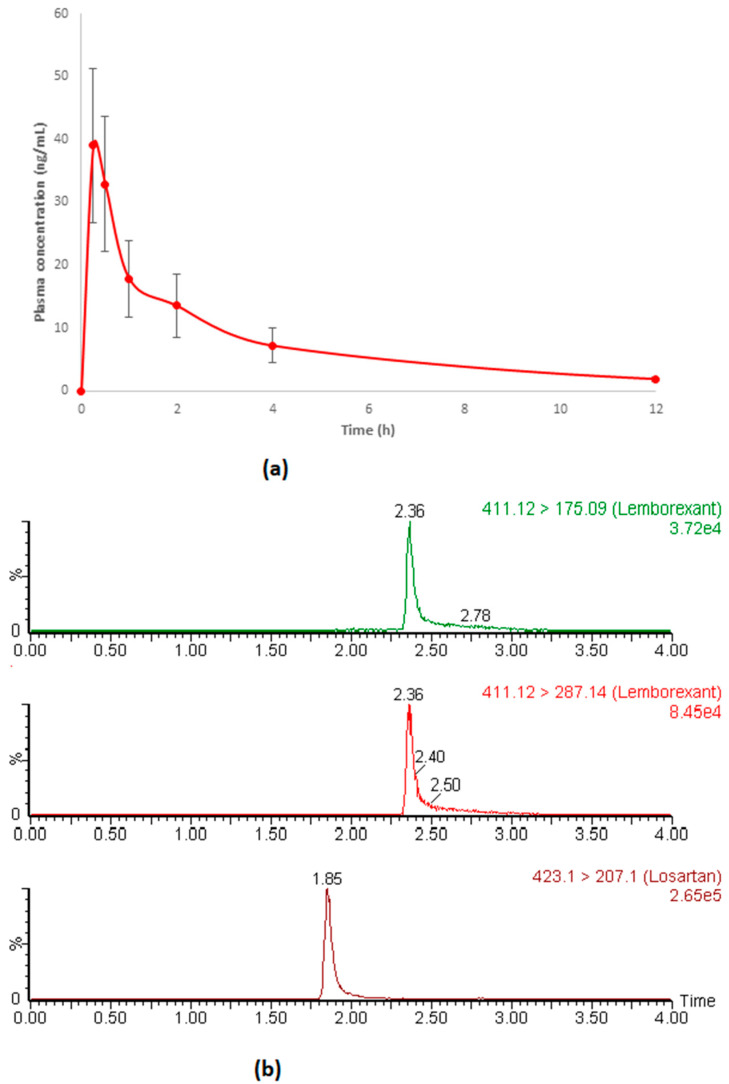
Plasma concentration versus time profile of LEM (**a**) and representative MRM chromatogram of LEM and IS (**b**) in rat after oral administration of 10 mg/kg LEM. [411.12 > 175.09 → Qualifier ion; 411.12 > 287.14 → Quantifier ion; 423.1 > 207.1 → IS].

To ensure the reliability of the assay, the validated method was applied in pharmacokinetic study of LEM in rats. Usually, human plasma is a unique matrix, which can be considered to analyze samples from other species, e.g., rats also. Although a slight difference is sometimes noted in the matrices among different species in the case of analysis by using TQD, a cross-validation in term of precision and bias was also performed in rat plasma matrix to ensure the robustness of the method. The results of basic pharmacokinetic parameters are presented in Table 6. The mean value of C_max_ and AUC_0–24 h_ of 39.69 ng/mL and 109.16 ng.h/mL, respectively, were achieved after intragastrical administration of 10 mg/kg of LEM. The elimination T½ and MRT value were 3.78 h and 4.41 h, respectively. These results are comparable to previously reported data of innovator submitted in USFDA [5], which further ensure the reliability of the assay. The plasma concentration versus time profile and representative MRM chromatograms of LEM in rats are presented in Figure 4.

### 3.6. Limitation of the Study

The limitation of this study is that it not directly applied in real human plasma samples due to unavailability of approved formulation in kingdom. Upcoming studies are considered necessary to conduct application in human samples to ensure more reliability of the proposed assay.

## 4. Conclusions

A sensitive and reliable assay was developed and validated for the detection and quantification of LEM in plasma matrix. Being a drug of abuse, the assay was validated following the SWGTOX guidelines, which are able be to used for both detection and quantification of LEM in blood samples. All validation parameters were within the acceptable ranges and linear between the concentration range of 1–300 ng/mL, which is sufficient to detect intoxicating or fatal concentrations of LEM. The assay was successfully applied in pharmacokinetic study of LEM in rats. Moreover, the assay could be used for futuristic forensic toxicology testing, therapeutic drug monitoring, and pharmacokinetics drug interaction studies after conducting its application in real human samples.

## Figures and Tables

**Table 1:** Optimized UPLC-MS/MS parameters for LEM and IS.

Compound	t_R_ (min)	Q1 [M+H]^+^	CV (V)	Q3 [M+H]^+^	CE (eV)	dt (s)
Lemborexant	2.36	411.12	26	287.14	14	0.106
				175.09 *	28	0.106
IS	1.86	423.1	22	207.1	20	0.106

t_R_ = retention time; Q1 = precursor ion; CV = cone voltage; dt = dwell time; Q3 = product ion [M+H]^+^, CE = collision energy), * Qualifier ion.

**Table 2:** Gradient condition of mobile phase used for sample separation.

Time (min)	Flow (mL/min)	Solvent A	Solvent B	Curve
initial	0.3	80	20	
0.50	0.3	20	80	6
1.00	0.3	50	50	6
1.50	0.3	80	20	6
4.00	0.3	80	20	6

**Table 3:** Intra- and inter-day precision and bias data of LEM in human plasma samples.

Nominal QC (ng/mL)	Precision (RSD, %)		Bias (RE, %)	
	Intra-Day	Inter-Day	Intra-Day	Inter-Day
1.0	11.49	9.35	6.99	10.74
3.0	5.91	5.79	−10.03	−9.12
50	3.60	3.35	2.25	1.66
250	1.68	4.85	−8.77	−6.12

**Table 4:** Matrix effects and recovery percentage of LEM and IS in plasma (*n* = 6).

Compound	Nominal QC (ng/mL)	Matrix Effects		Extraction Recovery	
		% Mean	RSD, %	% Mean	RSD, %
LEM	3.0	106.8	2.95	79.1	6.54
	50	93.3	5.18	67.8	3.97
	500	87.4	9.65	74.7	9.49
Overall mean		95.8	10.4	73.9	5.68
IS	400	86.9	6.95	80.7	7.39

**Table 5:** Stability data of LEM in human plasma.

Stability	Nominal Concentration (ng/mL) (*n* = 6)	Precision (RSD, %)	BIAS (RE, %)
Bench top (8 h)			
	3.0	4.80	9.22
	250	6.75	−6.40
Freeze thaw (3 cycle)			
	3.0	8.15	12.11
	250	−5.56	−3.93
Auto-sampler (24 h)			
	3.0	6.41	2.06
	250	9.07	4.87
60 days at −80 °C			
	3.0	5.13	−6.78
	250	9.53	−9.73

**Table 6:** Pharmacokinetic parameters of LEM in male rats (*n* = 6) after single dose administration (10 mg/kg p. o.).

Pharmacokinetic Parameters	Unit	Values (mean ± SD)
C_max_	ng/mL	39.69 ± 11.69
T_max_	h	0.25
AUC_0−t_	ng.h/mL	109.16 ± 21.06
T^1/2^	h	3.78 ± 1.07
Kel	h	0.19 ± 0.04
MRT	h^−1^	4.41 ± 1.0

## Data Availability

All the data generated from the study are clearly presented in the manuscript.
